# Development and Validation of a Tool to Measure Patient Assessment of Clinical Compassion

**DOI:** 10.1001/jamanetworkopen.2019.3976

**Published:** 2019-05-17

**Authors:** Brian W. Roberts, Michael B. Roberts, Jady Yao, Joshua Bosire, Anthony Mazzarelli, Stephen Trzeciak

**Affiliations:** 1 Department of Emergency Medicine, Cooper University Health Care, Cooper Medical School of Rowan University, Camden, New Jersey; 2 Center for Humanism, Cooper Medical School of Rowan University, Camden, New Jersey; 3 Institutional Research and Outcomes Assessment, Philadelphia College of Osteopathic Medicine, Philadelphia, Pennsylvania; 4 Department of Patient Family Centered Care, Cooper University Health Care, Camden, New Jersey; 5 Department of Medicine, Cooper University Health Care, Cooper Medical School of Rowan University, Camden, New Jersey

## Abstract

**Importance:**

Clinician compassion is a vital element of health care quality. Currently, there appears to be no validated and feasible method for health care organizations to measure patient assessment of clinician compassion on a large scale.

**Objective:**

To develop and validate a tool for measuring patient assessment of clinician compassion that can be used in conjunction with the Clinician and Group Consumer Assessment of Healthcare Providers and Systems (CG-CAHPS) survey.

**Design, Setting, and Participants:**

This prospective cohort study took place from June 1 to August 30, 2018, at a US academic health care system among a pilot cohort consisting of 3325 adult patients and a validation cohort consisting of 3483 adult patients, both of whom had an outpatient clinic visit and completed the CG-CAHPS survey.

**Main Outcomes and Measurements:**

After a comprehensive literature review, 12 candidate survey items were developed. Face and construct validity were performed. Candidate items were disseminated to patients in conjunction with the CG-CAHPS survey in a series of 2 studies: (1) exploratory factor analysis in one cohort to determine the factor structure and the most parsimonious set of items; and (2) validity testing in a second cohort using confirmatory factor analysis. Reliability was tested using Cronbach α. Convergent validity was tested with patient assessment of clinician communication and overall satisfaction questions from CG-CAHPS survey.

**Results:**

Overall, 6493 patient responses were analyzed. The mean (SD) age was 60 (15) years, 4239 patients (65.3%) were women, and 5079 (78.2%) were white. Exploratory factor analyses identified a 5-item compassion measure to be the most parsimonious. Confirmatory factor analyses found good fit. The compassion measure demonstrated good internal consistency (α = 0.94) and convergent validity (clinician communication: ρ = 0.44; overall satisfaction: ρ = 0.52) but reflected a patient experience domain (compassionate care) distinct from what is currently captured in the CG-CAHPS survey.

**Conclusions and Relevance:**

A simple 5-item tool to measure patient assessment of clinician compassion was developed and validated for use in conjunction with CG-CAHPS survey.

## Key Points

**Question:**

Can patient perception of clinician compassion be measured on a large scale across health care organizations?

**Findings:**

In this cohort study of 6493 patients, a 5-item tool to measure patient assessment of clinician compassion was developed and validated for use in conjunction with the Clinician and Group Consumer Assessment of Healthcare Providers and Systems survey. The compassion measure demonstrated good internal consistency and convergent validity and reflected a patient experience domain (compassion) distinct from what the Clinician and Group Consumer Assessment of Healthcare Providers and Systems survey captures.

**Meaning:**

The 5-item compassion measure appears to be a reliable tool to measure patient perception of clinician compassion on a large scale.

## Teaser

This cohort study describes the development and validation of a 5-item tool for measuring patient assessments of clinician compassion that can be used in conjunction with the Clinician and Group Consumer Assessment of Healthcare Providers and Systems (CG-CAHPS) survey.

## Introduction

Compassion is the emotional response to another’s pain and suffering involving an authentic desire to help.^1,2,3^ In health care, compassion is a fundamental component of the therapeutic relationship, and patients and physicians consider compassion to be a vital element of high-quality care.^4^ Not only is compassion desired by patients, but compassion is also associated with better clinical outcomes across numerous conditions.^5^ For example, greater patient perception of clinician compassion has been associated with reduced migraine headache disability,^6^ enhanced immune response, shorter duration and lower severity of cold symptoms,^7^ reduced depression and better quality of life,^8,9,10^ better patient-reported outcome measures among trauma patients,^11^ and better patient adherence to prescribed therapy.^12^ A randomized clinical trial among oncology patients^13^ found a compassion intervention to significantly reduce patient anxiety. In addition, focus on compassionate care is of potential importance to the financial sustainability of health care organizations, given that a lack of compassion is associated with increased resource utilization and health care spending.^14,15,16,17^

Given that patient perception of compassionate care is associated with patient outcomes and health care costs, having the ability to measure patient assessment of compassion (as opposed to self-assessment of compassion by the clinician or assessment of compassion by a third-party observer) is of the utmost importance for health care organizations. Although several compassion and empathy measurement tools have been tested in prior research studies,^18,19,20^ to our knowledge, no measurement tool has been designed or validated for dissemination on a large scale to heterogeneous populations of patients. To our knowledge, no previous compassion measurement tool has been formatted and is brief enough to be administered with the Clinician and Group Consumer Assessment of Healthcare Providers and Systems (CG-CAHPS) survey, the patient satisfaction survey for adult outpatient clinic visits used by the US Centers for Medicare & Medicaid Services for all health care organizations that receive payments from Medicare.^21^ Having a compassion measurement tool that can be distributed to patients through the CG-CAHPS mechanism across entire health care organizations would be an important advance for measuring this important aspect of health care quality. The objectives of this study were to develop and validate a simple compassion measurement tool, which can be incorporated into and disseminated with the CG-CAHPS survey.

## Methods

### Setting

This study was conducted across an academic health care system in the United States (Cooper University Health Care, Camden, New Jersey). The study took place from June 1 to August 30, 2018. Given this research involved only survey procedures and information was recorded in such a manner that participants could not be identified, the institutional review board at Cooper University Health Care considered this study exempt from 45 Code of Federal Regulations requirements.^22^ This study is reported in accordance with the Strengthening the Reporting of Observational Studies in Epidemiology (STROBE) reporting guideline for cohort studies.^23^

### Study Population

The study population consisted of patients 18 years and older who had an outpatient clinic visit and completed the CG-CAHPS survey. We matched our inclusion criteria to correspond with the CG-CAHPS survey, given that a primary objective of this measurement tool is to work in conjunction with that survey. At Cooper University Health Care, the CG-CAHPS survey is sent to all patients who have an outpatient clinic visit and have a mailing address or email on file. Patients are excluded from receiving the survey if they already received it for a visit with the same specialty in the past 90 days. Patient visit data from the day before are sent to the survey vendor (Press Ganey Associates) each day. The vendor sends out surveys to patients within 48 hours of receiving the data. The vendor collates responses and returns the deidentified survey data to our institution.

### Clinician and Group Consumer Assessment of Healthcare Providers and Systems Survey

The CG-CHAPS survey is a validated and reliable measurement tool, which was developed to elicit reports from patients regarding their health care experiences, including measurement of clinician communication skills and overall patient satisfaction.^21^ It has undergone extensive psychometric testing. It has been found to be very reliable for clinician communication skills (Cronbach α = 0.88).^21^

### Candidate Item Development

We first derived a theoretical understanding of the construct of compassion by performing a systematic review of the published health care literature focused on (1) the conceptual basis of compassion and (2) mechanisms of action by which compassion can improve clinical and health care–related outcomes.^5^ Based on this literature, we defined compassion as an emotional response to another’s pain and suffering involving an authentic desire to help.^1,2,3^ We reviewed the most commonly used compassion measurement tools to look for overlap and important distinctions between tools.^13,18,19,20^ Based on our findings, we developed 12 candidate items for potential inclusion in the final compassion measure. Four experts in the field of compassionate patient care independently examined these items to ensure construct and face validity (eAppendix in the Supplement). In addition, since our goal was to allow for easy distribution to large populations, the candidate items were reviewed by 2 experienced patient experience analysts as well as the research team from Press Ganey Associates (which partners with most US hospitals in the administration and reporting of CG-CAHPS surveys), who provided feedback on items to ensure feasible dissemination with the CG-CAHPS survey.

### Pilot Testing

The 12 candidate items (eTable 1 in the Supplement) were incorporated into and distributed with the CG-CAHPS survey for a 30-day period. We performed exploratory factor analysis and examined factor loadings for adequate loading with the criterion of 0.40 or greater on a single factor.^24^ Exploratory factor analysis examines the underlying structure of the candidate items and tests if the results can be best explained by a single underlying construct or multiple underlying constructs. Prior to performing exploratory factor analysis, we performed the Kaiser-Meyer-Olkin measure of sampling adequacy (values >0.8 suggest sampling is adequate for factor analysis) and the Bartlett test of sphericity (*P* < .05 suggests factor analysis is useful for the data) to ensure use of factor analysis was appropriate.

Given that our goal was to develop a concise measurement tool that can be easily added to the CG-CAHPS survey for wide dissemination across all practice areas of a health care organization, we identified the candidate items with the strongest factor loadings on a single construct (ie, items with the strongest correlation to the underlying construct). We removed items with lower factor loadings and compared the Akaike information criterion and the Bayesian information criterion between the original model (ie, all 12 items) and the nested model (ie, items with the strongest factor loadings) to identify the most parsimonious model (ie, lower Akaike information criterion and Bayesian information criterion).

### Statistical Analysis

After the pilot testing, a set of 5 survey questions was identified as the most parsimonious set for final validation. Prior to validation testing, these final items underwent minor, nonsubstantive wording modification based on feedback from our experts in the field of compassionate patient care and patient experience. The 5-item compassion measure was then incorporated into and distributed with the CG-CAHPS for a second 30-day period.

We performed confirmatory factor analysis (using structural equation modeling) on the final 5 items and calculated standardized coefficients. Confirmatory factor analysis tests how correctly a hypothesized model (in this case, a theorized single construct) matches the observed data. We examined fit indices (which take into account total sample size), including comparative fit index (CFI), Tucker-Lewis Index (TLI), and standardized root mean squared residual (SRMR). We defined our model as having good fit if CFI > 0.95, TLI > 0.95, and SRMR < 0.08.^25^ We chose to examine fit indices because, when the sample size is large, a χ^2^ test for model fit is often significant (ie, α > .05), suggesting that model is a poor fit, even when the model is, in practice, a good fit.^26,27^

We obtained a composite score for the 5-item compassion measure by summing the scores for each item. Using Spearman correlation coefficients, we tested convergent validity between the 5-item compassion measure total score and (1) physician communication in CG-CAHPS questions (ie, physician’s ability to listen, explain, give instructions, show respect, know medical history, and spend enough time with patient) and (2) overall patient satisfaction in CG-CAHPS. We expected the 5-item compassion measure to have a positive correlation with yet be distinct from physician communication and overall satisfaction. To determine whether the compassion measure formed a discrete construct or whether it simply reflected the clinician’s ability to communicate with the patient, confirmatory factor analysis was used to test a single construct model (ie, the 5-item compassion measure and the CG-CAHPS communication questions loading on a single latent variable) as well as a 2-construct model (ie, the 5-item compassion measure and CG-CAHPS communication questions loading on separate latent variables). We further tested the null hypothesis that the covariance between the 2 latent structures is 1 (ie, single-construct model). We tested this hypothesis using a likelihood ratio test to compare 2 nested models: a model with covariance between the 2 latent models constrained at 1 (ie, single-construct model) vs a model with covariance between the 2 latent models allowed to be a free parameter (ie, 2-construct model).

Internal reliability was tested among the entire validation cohort as well as among individual specialties using Cronbach α. We also tested internal reliability among physicians only compared with physician assistants and advanced nurse practitioners.

Self-reported patient characteristics were described using mean and standard deviation or median and interquartile range for continuous variables based on the distribution of data and number and percentage for categorical variables. To assess for nonresponse bias, we performed separate ordered probit regression analyses to test if partial response (compared with complete response) to the 12 compassion measure items was associated with the individual item scores among the pilot cohort (eg, test if nonresponse to any of the items 2-12 predicted the score of item 1). In addition, we performed separate ordered probit regression analyses to test if partial response (compared with complete response) to the 5-item compassion measure was associated with the individual items among the validation cohort (eg, test if a nonresponse to any of the items 2-5 predicted the score of item 1). *P* values less than .05 were considered statistically significant, and tests were 2-tailed. All data were exported into Stata version 15.1 (StataCorp) for analysis.

## Results

### Patients

During the pilot testing phase, 21 732 surveys were delivered, and we received 3325 responses (15.3%). Overall, 294 surveys had a partial response and were excluded from analyses, resulting in 3031 (13.9%) completed responses, assessing 313 different clinicians across more than 15 specialties, including primary care, surgery, gynecology/obstetrics, cardiology, orthopedics, hematology/oncology, endocrinology, gastroenterology, pulmonary, urology, neurology, rheumatology, otolaryngology, nephrology, and anesthesiology. During the validation phase for the final 5-item compassion measure, 23 066 surveys were delivered, and we received 3483 responses (15.1%). Of these, 21 surveys had a partial response and were excluded from analyses, resulting in 3462 complete measurement responses (15.0%), assessing 312 different clinicians across the specialties listed earlier. The partial response rate for the compassion measure items among returned surveys was lower among the validation cohort compared with the pilot cohort (pilot cohort: 294 of 3325 [8.8%]; validation cohort: 21 of 3483 [0.6%]). We did not find evidence of nonresponse bias for individual items among either cohort (eTable 2 and eTable 3 in the Supplement).

Table 1 displays patient characteristics for the pilot and validation testing groups. The age ranged from 18 to 101 years among both groups; the mean (SD) age was 60 (15) years. Overall, 4239 of 6493 patients (65.3%) were women, 5079 (78.2%) were white, and 4498 (69.2%) had at least some college education.

**Table 1.** Patient Characteristics

Characteristic	Cohort, No. (%)	
	Pilot (n = 3031)	Validation (n = 3462)
Age, mean (SD), y	60 (15)	59 (15)
Women	2010 (66.3)	2229 (64.4)
Race/ethnicity		
White	2390 (78.9)	2689 (77.7)
Black/African American	359 (11.8)	396 (11.4)
Asian American	86 (2.8)	109 (3.1)
Hawaiian or other Pacific Islander	4 (0.1)	9 (0.3)
American Indian or Alaska Native	27 (0.9)	37 (1.1)
Other	124 (4.1)	168 (4.9)
Hispanic or Latino descent	146 (4.8)	211 (6.1)
Highest education level completed		
≤8th Grade	44 (1.5)	47 (1.4)
Some high school	75 (2.5)	105 (3.0)
High school graduate	740 (24.1)	892 (25.8)
Some college	906 (29.9)	1052 (30.4)
4-y College graduate	545 (18.0)	609 (17.6)
>4 y College	679 (22.4)	707 (20.4)
Unknown	42 (1.4)	50 (1.4)
Visit to usual clinician	1702 (56.2)	1837 (53.1)
Test ordered by clinician in last 3 mo	1531 (50.1)	1765 (51.0)
Perceived wait time in examination room, median (IQR), min	7 (5-12)	6 (5-11)

Abbreviation: IQR, interquartile range.

### Pilot Testing

We found our data were appropriate for factor analysis (Kaiser-Meyer-Olkin, 0.96; Bartlett test of sphericity, *P* < .001). Factor analysis found all 12 items loaded well on a single construct (all factor loadings >0.65). We found 5 items resulted in the most parsimonious model (Box). Removal of any of the remaining items did not meaningfully improve model fit.

Box. **Final Items of the 5-Item Compassion Measure^a^**

1. How often do you feel your provider^b^ cares about your emotional or psychological well-being?
2. How often do you feel your provider is interested in you as a whole person?
3. How often do you feel your provider is considerate of your personal needs?
4. How often do you feel your provider is able to gain your trust?
5. How often do you feel your provider shows you care and compassion?

^a^Each item response scaled as 1, never; 2, sometimes; 3, usually; 4, always.

^b^Provider refers to physicians, advanced nurse practitioners, or physician assistants.

### Validation and Reliability

Confirmatory factor analysis among the validation cohort found our final 5 items loaded well on a single construct (all standardized coefficients >0.80). We found our model had good fit based on our definition (CFI = 0.98; TLI = 0.95; SRMR = 0.02). As expected, the χ^2^ test for model fit was significant. Internal reliability remained excellent (Cronbach α = 0.94) among the entire validation cohort as well as across specialties (α > 0.90) (Table 2).

**Table 2.** Cronbach α for 3462 Responses to the 5-Item Compassion Measure by Medical Specialty

Specialty	Responses, No. (%)	Cronbach α
Primary care	925 (26.7)	0.94
Surgery	349 (10.1)	0.94
Gynecology/obstetrics	312 (9.0)	0.95
Cardiology	292 (8.4)	0.94
Orthopedics	286 (8.3)	0.95
Hematology/oncology	269 (7.8)	0.92
Endocrinology	215 (6.2)	0.94
Gastroenterology	126 (3.6)	0.91
Pulmonary	112 (3.2)	0.93
Urology	101 (2.9)	0.94
Neurology	99 (2.9)	0.95
Rheumatology	67 (1.9)	0.95
Otolaryngology	77 (2.2)	0.94
Nephrology	47 (1.4)	0.93
Anesthesiology	64 (1.8)	0.94
Other	121 (3.5)	0.94
Physicians only (all specialties)	3231 (93.3)	0.94
Advanced nurse practitioners or physician assistants (all specialties)	231 (6.7)	0.94

The 5-item compassion measure had a moderate correlation with clinician communication (ρ = 0.44; *P* < .001) and overall patient satisfaction (ρ = 0.52; *P* < .001). Using confirmatory factor analysis, we found a single-factor model (the 5-item compassion measure and the CG-CAHPS communication questions loaded on a single latent variable) to have poor fit (CFI = 0.77; TLI = 0.71; SRMR = 0.13); however, we found a 2-factor model (5-item compassion measure and CG-CAHPS communication questions loaded on separate latent variables) to have good fit (CFI = 0.97; TLI = 0.96; SRMR = 0.04). The likelihood ratio test comparing 2 nested models was significant; therefore, we rejected the null hypothesis that the covariance between the 2 latent structures is 1 (ie, the 2-factor model has better fit).

The 5-item compassion measure ranged the full scale (5-20). Consistent with trends in patient satisfaction data in the United States, most responses were favorable (ie, negatively skewed).^28^ Table 3 displays frequencies of responses for each item; frequencies of response for the CG-CAHPS communication and overall patient satisfaction questions are available in eTable 4 and eTable 5 in the Supplement. We found a high proportion of responses were perfect scores for physician communication and overall patient satisfaction (physician communication: 2788 of 3165 [88.1%]; overall patient satisfaction: 2723 of 3453 [78.9%]). Comparatively, we found that fewer respondents gave perfect scores for the 5-item compassion measure (2471 of 3462 [71.3%]).

**Table 3.** Response Frequencies for 3462 Responses to the 5-Item Compassion Measure Among the Validation Cohort

5-Item Compassion Measure^a^	Responses, No. (%)			
	1, Never	2, Sometimes	3, Usually	4, Always
1. How often do you feel your provider cares about your emotional or psychological well-being?	49 (1.4)	164 (4.7)	570 (16.5)	2679 (77.4)
2. How often do you feel your provider is interested in you as a whole person?	37 (1.1)	138 (4.0)	486 (14.0)	2801 (80.9)
3. How often do you feel your provider is considerate of your personal needs?	33 (1.0)	120 (3.5)	474 (13.7)	2835 (81.9)
4. How often do you feel your provider is able to gain your trust?	28 (0.8)	101 (2.9)	504 (14.6)	2829 (81.7)
5. How often do you feel your provider shows you care and compassion?	22 (0.6)	86 (2.5)	394 (11.4)	2960 (85.5)

^a^ Provider refers to physicians, advanced nurse practitioners, or physician assistants.

## Discussion

This study provides the initial validation of a brief, simple measurement tool to assess patient perception of clinician compassion. We found the 5-item compassion measure can be easily combined with the CG-CAHPS survey for distribution across health care organizations. The 5-item compassion measure was found to be very reliable, as demonstrated by the consistently high α across specialties. As expected, we found the measure to have only a moderate correlation with the CG-CAHPS physician communication and overall patient satisfaction scores, suggesting the compassion measure is not a redundant patient satisfaction measure. In addition, based on our confirmatory factor analysis results, although there appears to be overlap between compassion and effective communication, the compassion measure was found to assess a separate construct. These results suggest that the compassion measure is a specific measure of patient perception of compassion and that compassion involves more than simply listening and explaining medical details. Further research is required to determine what other clinician behaviors are associated with patient perception of compassion.

Although prior studies using compassion and empathy measurement tools exist,^18,19,20^ development of the 5-item compassion measure is novel in that it is a patient assessment of clinician compassion as opposed to a third-party observation or self-assessment. This is important because it is likely the patients’ experience that drives the association of compassion with clinical outcomes. Further, the scale is brief and simple, allowing it to be easily disseminated with the CG-CAHPS for large-scale assessment of clinician compassion across health care organizations.

Ease of dissemination is of the utmost importance. Evidence suggests that compassion in health care is suboptimal worldwide.^5^ This is a significant public health issue given the effect that compassion (or lack thereof) can have on health care outcomes. First, there is an abundance of evidence supporting the association of compassionate, patient-centered care with better clinical outcomes for patients.^5,6,7,8,9,10,11,12^ Alternatively, a lack of clinician compassion is associated with lower quality of care and increased risk of harm to patients through medical errors.^29^ Second, a lack of compassion in health care systems is associated with increased resource utilization and health care spending. These increased costs are likely secondary to lower quality of care along with inadequate human connections between clinicians and patients, resulting in clinicians being more reliant on (often unnecessary) testing, technologies, and referrals to specialists.^14,15^ Third, a lack of compassion has been associated with clinician burnout, which has a major economic toll on health care systems as well as the health of patients.^29,30^ There is currently evidence that increased clinician compassion is associated with more resilience, improved well-being, and decreased burnout among clinicians.^31,32,33^

It is now clear that being compassionate is not simply an inherent trait, which clinicians either do or do not possess, but rather that compassionate behaviors can in fact be taught and learned.^2,34^ Thus, by being able to measure compassion on a large scale across health care organizations, it will be possible to rapidly identify physicians who are not perceived as being compassionate by their patients, and interventions to increase compassion can be considered. In addition, the 5-item compassion measure has implications for research. The use of an easily distributed, validated measurement tool for assessing patient perception of compassion will allow for rigorous testing of interventions aimed at increasing health care compassion and improving subsequent long-term outcomes (both clinical and economic) across numerous large-scale studies, using various clinical protocols, among different patient populations.

### Limitations

We acknowledge that this study has important limitations to consider. First, this study was performed in a single health care system; thus, it is possible that a study performed among a different population would find different results. However, our consistent reliability across 15 different specialties suggests potential generalizability. Second, the 5-item compassion measure scores were skewed toward high compassion (ie, 71.3% perfect scores). However, the commonly used CG-CAHPS items allowed for even less variability; physician communication received 88.1% perfect scores and overall satisfaction received 78.9% perfect scores. Third, although we based the development of our measurement items on previous literature, which incorporated patient opinion on compassion as well as patient input on previous measurement tools, we did not directly involve patients in the development of the measure. Fourth, although we did not find partial response to be a predictor of any of the compassion measure items in the pilot or validation cohorts, there remains the possibility of nonresponse bias. Further research is required to test if response rate is correlated with scores. The overall low survey response rate in this study is consistent with low response rates seen in previous studies involving the administration of CG-CAHPS.^35,36^ Of note, by decreasing the number of compassion measure items from 12 to 5, the partial response rate decreased by 8.2%. This suggests that using a parsimonious measure may improve response rates compared with a longer measure.

## Conclusions

The 5-item compassion measure appears to be a reliable tool to measure patient perception of clinician compassion on a large scale. Further testing among varying cohorts is warranted to further test generalizability of this measurement tool.

## Notes

Supplement. **eAppendix.** Compassionate Care Construct and Face Validity Expert Reviewers **eTable 1.** Initial 12 Candidate Items That Underwent Pilot Testing **eTable 2.** Separate Ordered Probit Regression Models With Partial Response for 12-Item Compassion Measure **eTable 3.** Separate Ordered Probit Regression Models With Partial Response for 5-Item Compassion Measure **eTable 4.** Response Frequencies for Clinician and Group Consumer Assessment of Healthcare Providers and Systems Survey Communication Questions Among Validation Cohort **eTable 5.** Response Frequencies for Clinician and Group Consumer Assessment of Healthcare Providers and Systems Survey Overall Patient Satisfaction Question Among Validation Cohort
