# Supplementary material for: Development and Validation of a Tool to Measure Patient Assessment of Clinical Compassion
Source: JAMA Netw Open. 2019 May 17;2(5):e193976. doi: 10.1001/jamanetworkopen.2019.3976 (PMC6537812; doi:10.1001/jamanetworkopen.2019.3976)
Supplement: Supplement. — eAppendix. Compassionate Care Construct and Face Validity Expert Reviewers eTable 1. Initial 12 Candidate Items That Underwent Pilot Testing eTable 2. Separate Ordered Probit Regression Models With Partial Response for 12-Item Compassion Measure eTable 3. Separate Ordered Probit Regression Models With Partial Response for 5-Item Compassion Measure eTable 4. Response Frequencies for Clinician and Group Consumer Assessment of Healthcare Providers and Systems Survey Communication Questions Among Validation Cohort eTable 5. Response Frequencies for Clinician and Group Consumer Assessment of Healthcare Providers and Systems Survey Overall Patient Satisfaction Question Among Validation Cohort [file jamanetwopen-2-e193976-s001.pdf]

Supplementary Online Content

Roberts BW, Roberts MB, Yao J, Bosire J, Mazzarelli A, Trzeciak S. Development and validation of a tool to measure patient assessment of clinical compassion. *JAMA Netw Open*. 2019;2(5):e193976. doi:10.1001/jamanetworkopen.2019.3976

eAppendix. Compassionate Care Construct and Face Validity Expert Reviewers

eTable 1. Initial 12 Candidate Items That Underwent Pilot Testing

eTable 2. Separate Ordered Probit Regression Models With Partial Response for 12-Item Compassion Measure

eTable 3. Separate Ordered Probit Regression Models With Partial Response for 5-Item Compassion Measure

eTable 4. Response Frequencies for Clinician and Group Consumer Assessment of Healthcare Providers and Systems Survey Communication Questions Among Validation Cohort

eTable 5. Response Frequencies for Clinician and Group Consumer Assessment of Healthcare Providers and Systems Survey Overall Patient Satisfaction Question Among Validation Cohort

This supplementary material has been provided by the authors to give readers additional information about their work.

© 2019 Roberts BW et al. *JAMA Network Open.*

eAppendix. Compassionate Care Construct and Face Validity Expert Reviewers

1) Edward Viner, MD. Founder and Director of the Center for Humanism, Cooper Medical School of Rowan University, Camden, NJ.

2) Mark Angelo, MD. Head of Palliative Care, Cooper University Health Care, Camden, NJ.

3) Stephen Trzeciak, MD, MPH. Professor and Chair of Medicine, Cooper Medical School of Rowan University, Camden, NJ. Author of book about compassion science, entitled: "Compassionomics."

4) Terry Ricca, RN. Chief Patient Experience Officer, Cooper University Health Care, Camden, NJ.

© 2019 Roberts BW et al. *JAMA Network Open.*

eTable 1. Initial 12 Candidate Items That Underwent Pilot Testing

**Initial items***
1. How often does your provider** present information to you in an understandable way?
2. How often does your provider listen to you and show interest in understanding your emotional needs?
3. How often does your provider express empathy, caring, and compassion about your emotional or psychological well-being?
4. How often is your provider considerate of your personal needs?
5. How often is your provider considerate of the effect of your current medical and/or emotional difficulties on your family and friends?
6. How often does your provider demonstrate sympathy when discussing your condition?
7. How often do you feel your provider is able to gain your trust?
8. How often do you feel your provider provided you with direction?
9. How often do you feel your provider spoke to you in a relaxed, appropriate tone of voice?
10. How often does your provider let you tell your "story"?
11. How often do you feel your provider is interested in your as a whole person?
12. How often does your provider show you care and compassion?

* Each item response scaled as 1=never; 2=sometimes; 3=usually; 4=always.

** Provider refers to physicians, advanced nurse practitioners, or physician assistants.

© 2019 Roberts BW et al. *JAMA Network Open.*

eTable 2. Separate Ordered Probit Regression Models With Partial Response for 12-Item Compassion Measure

Defined as patient missing 1 or more of the 12 compassion measure responses (yes/no), as the independent variable and each of the individual 12 compassion measure item scores as the dependent variables, among the pilot cohort (e.g. test if a non-response to any of the items 2-12 predict the score to item 1).

**Compassion measure item (dependent variable)**	**Completed response (n)**	**β**	**95 % CI**	**p-value**
How often does your provider present information to you in an understandable way?	3325	-0.052	-0.240 to 0.135	0.584
How often does your provider listen to you and show interest in understanding your emotional needs?	3325	-0.037	-0.222 to 0.174	0.691
How often does your provider express empathy, caring, and compassion about your emotional or psychological well-being?	3284	-0.044	-0.235 to 0.147	0.651
How often is your provider considerate of your personal needs?	3283	-0.003	-0.201 to 0.196	0.979
How often is your provider considerate of the effect of your current medical and/or emotional difficulties on your family and friends?	3189	-0.146	-0.358 to 0.066	0.178
How often does your provider demonstrate sympathy when discussing your condition?	3247	0.006	-0.196 to 0.208	0.955
How often do you feel your provider is able to gain your trust?	3286	0.003	-0.189 to 0.195	0.977
How often do you feel your provider provided you with direction?	3293	-0.069	-0.255 to 0.118	0.472

© 2019 Roberts BW et al. *JAMA Network Open.*

How often do you feel your provider spoke to you in a relaxed, appropriate tone of voice?	3302	0.118	-0.134 to 0.371	0.359
How often does your provider let you tell your “story”?	3325	-0.033	-0.216 to 0.149	0.894
How often do you feel your provider is interested in your as a whole person?	3309	0.003	-0.181 to 0.187	0.971
How often does your provider show you care and compassion?	3301	0.181	-0.038 to 0.399	0.105

© 2019 Roberts BW et al. *JAMA Network Open.*

eTable 3. Separate Ordered Probit Regression Models With Partial Response for 5-Item Compassion Measure

Defined as patient missing one or more of the 5-item compassion measure responses (yes/no), as the independent variable and each of the individual 5-item compassion measure item scores as the dependent variables, among the validation cohort (e.g. test if a non-response to any of the items 2-5 predict the score to item 1).

5-item compassion measure item (dependent variable)	Completed response (n)	β	95 % CI	p-value
How often do you feel your provider cares about your emotional or psychological well-being?	3483	-0.112	-0.661 to 0.436	0.688
How often do you feel your provider is interested in you as a whole person?	3473	-0.347	-0.107 to 0.377	0.347
How often do you feel your provider is considerate of your personal needs?	3483	-0.216	-0.771 to 0.338	0.444
How often do you feel your provider is able to gain your trust?	3483	-0.435	-0.956 to 0.085	0.101
How often do you feel your provider shows you care and compassion?	3471	-0.475	-1.29 to 0.341	0.254

© 2019 Roberts BW et al. *JAMA Network Open.*

eTable 4. Response Frequencies for Clinician and Group Consumer Assessment of Healthcare Providers and Systems Survey Communication Questions Among Validation Cohort

**CG-CAHPS Communication**	**1 (no)**	**3 (yes, sometimes)**	**4 (yes, definitely)**
1. Did this provider explain things in a way that was easy to understand?	20	116	3312
2. Did this provider listen carefully to you?	23	103	3323
3. Did this provider give you easy to understand information about these health questions or concerns?	27	122	3048
4. Did this provider seem to know the important information about your medical history?	55	253	3145
5. Did this provider show respect for what you had to say?	25	64	3362
6. Did this provider spend enough time with you?	35	102	3314

© 2019 Roberts BW et al. *JAMA Network Open.*

eTable 5. Response Frequencies for Clinician and Group Consumer Assessment of Healthcare Providers and Systems Survey Overall Patient Satisfaction Question Among Validation Cohort

Overall Patient Satisfaction	***0***	***1***	***2***	***3***	***4***	***5***	***6***	***7***	***8***	***9***	***10***
Using any number from 0 to 10, where 0 is the worst provider possible and 10 is the best provider possible, what number would you use to rate this provider?	13	6	4	11	6	25	9	45	188	423	2723

© 2019 Roberts BW et al. *JAMA Network Open.*
